# Who is screened for perinatal mental health? findings from an academic health system in California

**DOI:** 10.1007/s00737-026-01719-w

**Published:** 2026-05-09

**Authors:** Rebecca Woofter, Rashmi Rao, Misty Richards, May Sudhinaraset

**Affiliations:** 1 Department of Health Behavior, Society, and Policy, Rutgers, The State University of New Jersey, Piscataway, USA; 2 Department of Community Health Sciences, University of California, Los Angeles, Los Angeles, USA; 3 Department of Obstetrics and Gynecology, University of California, Los Angeles, Los Angeles, USA; 4 Department of Psychiatry, University of California, Los Angeles, Los Angeles, USA

**Keywords:** mental health screening, Edinburgh Postnatal Depression Scale, pregnancy, postpartum, perinatal

## Abstract

**Purpose:**

To identify sociodemographic characteristics, prenatal healthcare utilization, and health factors associated with completed mental health screenings during pregnancy and postpartum, respectively.

**Methods:**

We examined electronic medical records (EMR) data on documented perinatal mental health screenings from over 6000 patients who delivered singleton infants within one academic healthcare system in California between 2021 and 2023. Dependent variables included having at least one Edinburgh Postnatal Depression Scale (EPDS) score documented in the EMR during (1) pregnancy and (2) postpartum, among those who completed postpartum care within 12 weeks of delivery. We used univariate frequencies to determine rates of completed screenings and logistic regression models to identify patient characteristics associated with completed screenings in each time period.

**Results:**

Overall, 67% of patients completed screenings in pregnancy and 56% completed screenings in postpartum. In pregnancy, patients who were White, multiparous, did not prefer English, began prenatal care in the third trimester, and completed 10 or fewer prenatal care visits each had lower odds of completed screenings than their counterparts. In postpartum, patients who were White, privately insured, had vaginal deliveries, and had term births (at least 37 weeks of gestation) each had lower odds of completed screenings than their counterparts.

**Conclusions:**

Although California law requires perinatal mental health screening in pregnancy and postpartum , the identified inequities in screening suggest additional efforts are needed to ensure all perinatal patients are screened for mental health in accordance with the law. These efforts should be tailored to the populations least likely to be screened in order to achieve universal perinatal mental health screening.

**Supplementary Information:**

The online version contains supplementary material available at 10.1007/s00737-026-01719-w.

## Article highlights

• We found that 67% of patients had documented mental health screenings in pregnancy and 56% had screenings in postpartum.

• Screenings were associated with sociodemographic characteristics, prenatal healthcare utilization, and health factors.

• Additional efforts are needed to ensure all patients have documented screenings in both pregnancy and postpartum.

**Supplementary Information:**

The online version contains supplementary material available at 10.1007/s00737-026-01719-w.

## Introduction

Depression and anxiety are common in the perinatal period (during pregnancy and up and 12 months postpartum), each affecting 10–30% of patients (Dennis et al. 2017; Shorey et al. 2018; Bauman et al. 2020; Haight et al. 2024). Perinatal depression and anxiety are associated with adverse outcomes including preterm birth, cesarean deliveries, severe maternal morbidity, maternal and infant emergency department use, and infant mortality (Pluym et al. 2021; Brown et al. 2021, 2024; Simonovich et al. 2021; Zochowski et al. 2021; Abe et al. 2024; Rokicki 2024). Additionally, nearly one-quarter of all pregnancy-related deaths are attributed to mental health conditions (Trost et al. 2021; Chen et al. 2025).

The American College of Obstetricians and Gynecologists (ACOG) recommends that clinicians screen patients for mental health during pregnancy and postpartum to identify those with depression or anxiety symptoms and make appropriate referrals to mental healthcare (Committee on Clinical Practice Guidelines - Obstetrics 2023). However, studies suggest that 35% of patients are not screened during pregnancy and 15–36% are not screened during postpartum visits (Sidebottom et al. 2021; Interrante et al. 2022).

In addition to ACOG recommendations, several states mandate mental health screening during the perinatal period (Griffen et al. 2021), though few studies have examined screening following these state mandates. In 2007, New Jersey mandated mental health screening in the hospital after delivery. A study using 2009–2010 New Jersey birth records data found that 90% of patients had documented screenings during their delivery hospitalization (Farr et al. 2014). Additionally, even when states have not mandated perinatal mental health screening, some healthcare systems have instituted universal screening programs, with screening rates as high as 99% after implementation of these programs (Avalos et al. 2016; Miller et al. 2019; Johnson et al. 2021; Accortt et al. 2022).

In 2019, California implemented AB 2193 mandating that healthcare workers screen all patients for mental health during both prenatal and postpartum care. A prior analysis at one California healthcare system demonstrated that prenatal and postpartum screening rates increased after the mandate was implemented (Woofter et al. 2026). Even by 2023, though, less than 60% of patients were screened in both pregnancy and postpartum (Woofter et al. 2026). However, this study did not investigate which patients were screened versus not. It is important to identify which patients are not screened in the context of screening mandates, as additional policies or guidance may be needed to achieve universal screening and these efforts should tailored to the population not currently benefitting from the law.

Indeed, few studies to date have examined potential inequities in perinatal mental health screening. One study analyzed medical records data from 2016 at a Minnesota healthcare system and found that Black patients (vs. White), and those who did not prefer English, were younger, publicly insured, multiparous, and completed fewer prenatal care visits each had lower odds of perinatal mental health screenings compared to their counterparts (Sidebottom et al. 2021). Notably, this study was not in the context of a state screening mandate and data were collected 10 years ago. Another study analyzed medical records data from 2020 to 2023 at a California healthcare system after a universal screening program was implemented in 2017 for screening in the hospital after delivery (Woofter et al. 2025). While that study found that less than 2% of patients were not screened during hospitalization for delivery, patients who were Black (vs. White), publicly insured, multiparous, and had maternal and infant complications each had lower rates of screening than their counterparts (Woofter et al. 2025). However, these findings are limited in that only about 250 patients were not screened after the multi-phase quality improvement program for mental health screening during delivery hospitalization (Accortt et al. 2022). Additionally, this study focused on screening during hospitalization for delivery, rather than during outpatient prenatal or postpartum care.

Our study builds upon prior literature by using medical records data from 2021 to 2023 at an academic healthcare system in California with a diverse patient population to identify inequities in mental health screening during pregnancy and postpartum following implementation of a state screening mandate. In particular, we examine the sociodemographic characteristics, prenatal healthcare utilization, and health factors associated with completed screenings during pregnancy and postpartum after California implemented a screening mandate. Our study is the first to our knowledge to examine inequities in mental health screening during prenatal and postpartum care following a state screening mandate.

## Materials and methods

This study leverages de-identified electronic medical records (EMR) data for patients who delivered singleton infants within one academic healthcare system in Los Angeles, California between January 1, 2021 and December 31, 2023. This healthcare system provides prenatal care, labor and delivery, and postpartum care. For any patients who delivered twice in this period, one delivery was randomly sampled so that each patient is included only once in the dataset, resulting in a patient population of 6,928. This study was deemed exempt from review by the Institutional Review Board at the University of California, Los Angeles because it analyzed existing de-identified data. This project was deemed not human subjects research so did not involve informed consent.

### Measures

The two binary dependent variables were (1) at least one completed mental health screening documented in the EMR during pregnancy and (2) among those who completed at least one postpartum visit in the OBGYN department within 12 weeks of delivery, at least one completed mental health screening documented in the EMR within 12 weeks of delivery. In this healthcare system, patients are screened for perinatal mental health using the Edinburgh Postnatal Depression Scale (EPDS). The EPDS is a ten-item self-administered questionnaire that has been validated in pregnancy and postpartum to capture symptoms of depression and anxiety (Cox et al. 1987; Gibson et al. 2009; Levis et al. 2020; Smith-Nielsen et al. 2021).

The independent variables included sociodemographic characteristics, prenatal healthcare utilization, and health factors. These variables were selected based on previously identified factors associated with perinatal mental health screening, as well as known risk factors for perinatal depression or anxiety (Arafa and Dong 2019; Dekel et al. 2019; Bauman et al. 2020; Sidebottom et al. 2021; Yang et al. 2022; Logue et al. 2022; Bodunde et al. 2025; Woofter et al. 2025). Sociodemographic characteristics included self-reported race and ethnicity (non-Hispanic Asian/Pacific Islander (API), non-Hispanic Black, non-Hispanic Other/Multiracial, non-Hispanic White, and Hispanic), preferred language (English vs. non-English), age at delivery (< 25, 25–34, 35+), parity (primiparous vs. multiparous), and insurance status at delivery (private vs. public/none). Patients coded as Other/Multiracial included those who indicated their race was American Indian/Alaskan Native (which was too small to constitute a separate category), those who indicated “race not listed”, and those who selected multiple races but not Hispanic ethnicity. The categories for age, parity, and insurance status maintained as much detail as possible while minimizing small cell sizes in less common groups. Prenatal care (PNC) utilization was operationalized as the trimester in which PNC was initiated (first, second, third) and the total number of PNC visits completed in the OBGYN department (0–10, 11–20, 21–30, 31+).

Health factors included diagnosed hypertension and/or diabetes during pregnancy, diagnosed depression and/or anxiety during pregnancy, delivery type, and preterm birth (PTB). Patients were coded as having diagnosed hypertension and/or diabetes during pregnancy if they had any diagnoses in the EMR during pregnancy of hypertension and/or diabetes (see Appendix Table 4). Patients were coded as having diagnosed depression and/or anxiety during pregnancy if they had any diagnoses in the EMR during pregnancy of depression and/or anxiety (see Appendix Table 4). Each delivery was recorded in the EMR as vaginal or cesarean. Gestational age was noted in the EMR and PTB was coded as deliveries at fewer than 37 weeks of gestation.

### Analyses

First, univariate frequencies were used to characterize the patient population. Next, logistic regression models were conducted estimating the odds of completed screenings during pregnancy and postpartum, respectively, based on each relevant independent variable in separate models. Finally, logistic regression models were conducted estimating the odds of completed screenings during pregnancy and postpartum, respectively, including all relevant independent variables in one model. Independent variables for prenatal screening included sociodemographic characteristics and PNC utilization but not health factors, as these may have occurred after screening. Independent variables for postpartum screening included sociodemographic characteristics, PNC utilization, and health factors. Only patients who completed at least one postpartum visit in the OBGYN department within the first 12 weeks postpartum were included in the models for screenings during postpartum. Analyses were conducted using STATA 18/MP.

## Results

Table 1 shows the proportion of patients with completed prenatal and postpartum screenings as well as the frequencies of all independent variables. Of the initial 6,928 patients, 397 were missing data on race and ethnicity, resulting in a complete case sample of 6,531. Overall, 67.2% of patients completed prenatal screenings. Among the 5,590 patients who attended postpartum care within 12 weeks of delivery, 55.6% completed postpartum screenings. The most common racial and ethnic group was White (37.8%), followed by Hispanic (24.3%), API (21.1%), Other/Multiracial (11.2%), and Black (5.6%). Most patients preferred English (95.1%), were primiparous (62.4%), and had private insurance (81.4%). Nearly all patients were aged 25–34 (49.5%) or 35+ (46.5%) at delivery. Most patients began PNC in the first trimester (77.8%) and completed 11–20 PNC visits (61.4%). During pregnancy, 18.1% of patients had diagnosed hypertension and/or diabetes and 15.6% had diagnosed depression and/or anxiety. A total of 29.9% of deliveries were cesarean and 8.2% of patients had PTB.

**Table 1:** Patient characteristics in electronic medical records at an academic healthcare system in California (2021–2023; *N* = 6531)

Variable	Total *N* (%)
Screened during pregnancy	
No	2140 (32.8)
Yes	4391 (67.2)
Screened during postpartum (if attended 1 + postpartum visit, *N* = 5590)	
No	2484 (44.4)
Yes	3106 (55.6)
Race and ethnicity	
Non-Hispanic Asian/Pacific Islander	1378 (21.1)
Non-Hispanic Black	366 (5.6)
Non-Hispanic Other/Multiracial	731 (11.2)
Non-Hispanic White	2468 (37.8)
Hispanic	1588 (24.3)
Prefer English language	
Yes	6210 (95.1)
*No*	321 (4.9)
Age at delivery	
< 25	257 (3.9)
25–34	3235 (49.5)
35+	3039 (46.5)
Parity	
Primiparous	4072 (62.4)
Multiparous	2459 (37.7)
Insurance at delivery	
Private	5314 (81.4)
Public/None	1217 (18.6)
Trimester prenatal care initiated	
1st	5081 (77.8)
2nd	856 (13.1)
3rd	594 (9.1)
Number of prenatal visits	
0–10	595 (9.1)
11–20	4010 (61.4)
21–30	1693 (25.9)
31+	233 (3.6)
Hypertension and/or diabetes in pregnancy	
No	5351 (81.9)
Yes	1180 (18.1)
Depression and/or anxiety in pregnancy	
No	5514 (84.4)
Yes	1017 (15.6)
Delivery type	
Vaginal	4577 (70.1)
Cesarean	1954 (29.9)
Preterm birth	
No	5995 (91.8)
Yes	536 (8.2)

Table 2 shows the unadjusted and adjusted logistic regression models estimating the odds of completed prenatal screenings. Compared to White patients, API patients (AOR [adjusted odds ratio]: 1.27, 95%CI [confidence interval]: 1.09–1.46), Black patients (AOR 1.39, 95%CI: 1.08–1.78), and Hispanic patients (AOR 1.22, 95%CI: 1.05–1.41) each had higher odds of completed prenatal screenings. Those who did not prefer English (AOR 0.66, 95%CI: 0.51–0.84), were multiparous (AOR 0.59, 95%CI: 0.52–0.65), and who initiated PNC in the third trimester each had lower odds (AOR 0.78, 95%CI: 0.63–0.96) of completed prenatal screenings than their respective counterparts. Compared to those who completed 0–10 PNC visits, those who completed 11–20 PNC visits (AOR 1.41, 95%CI: 1.15–1.72), 21–30 PNC visits (AOR 1.94, 95%CI: 1.55–2.43), or 31 + PNC visits (AOR 2.93, 95%CI: 2.00-4.28) each had higher odds of completed prenatal screenings.

**Table 2:** Unadjusted and adjusted odds of completed screenings during pregnancy across all independent variables in electronic medical records at an academic healthcare system in California (2021–2023; *N* = 6531)

Variable	Unadjusted odds ratio (95% CI)	*P*-value	Adjusted odds ratio (95% CI)	*P*-value
Race and ethnicity				
Non-Hispanic Asian/Pacific Islander	1.23 (1.06–1.41)	0.005	1.27 (1.09–1.46)	0.002
Non-Hispanic Black	1.29 (1.01–1.64)	0.038	1.39 (1.08–1.78)	0.010
Non-Hispanic Other/Multiracial	1.09 (0.91–1.30)	0.335	1.18 (0.99–1.42)	0.067
Non-Hispanic White	Ref	-	Ref	-
Hispanic	1.05 (0.92–1.20)	0.494	1.22 (1.05–1.41)	0.008
Prefer English language				
Yes	Ref	-	Ref	-
No	0.57 (0.46–0.72)	< 0.001	0.66 (0.51–0.84)	0.001
Age at delivery				
< 25	1.02 (0.78–1.35)	0.870	1.02 (0.77–1.37)	0.868
25–34	Ref	-	Ref	-
35+	0.89 (0.80, 0.99)	0.035	0.95 (0.85–1.06)	0.347
Parity				
Primiparous	Ref	-	Ref	-
Multiparous	0.56 (0.50–0.62)	< 0.001	0.59 (0.52–0.65)	< 0.001
Insurance at delivery				
Private	Ref	-	Ref	-
Public/none	0.86 (0.76–0.99)	0.029	0.99 (0.85–1.16)	0.932
Trimester prenatal care initiated				
1st	Ref	-	Ref	-
2nd	0.89 (0.77–1.04)	0.154	1.03 (0.88–1.22)	0.691
3rd	0.56 (0.47–0.66)	< 0.001	0.78 (0.63–0.96)	0.020
Number of prenatal care visits				
0–10	Ref	-	Ref	-
11–20	1.65 (1.38–1.96)	< 0.001	1.41 (1.15–1.72)	0.001
21–30	2.35 (1.94–2.85)	< 0.001	1.94 (1.55–2.43)	< 0.001
31+	3.47 (2.42–4.98)	< 0.001	2.93 (2.00-4.28)	< 0.001

Table 3 shows the unadjusted and adjusted logistic regression models estimating the odds of completed postpartum screenings. Compared to White patients, API patients (AOR 1.18, 95%CI: 1.02–1.36) and Hispanic patients (AOR 1.34, 95%CI: 1.16–1.56) each had higher odds of completed postpartum screenings. Those with public insurance or no insurance (AOR 1.28, 95%CI: 1.09–1.51), those who had cesarean deliveries (AOR 1.24, 95%CI: 1.10–1.40), and those with PTB (AOR 1.26, 95%CI: 1.02–1.57) each had higher odds of completed postpartum screenings than their respective counterparts.

**Table 3:** Unadjusted and adjusted odds of completed screenings during postpartum among those who completed postpartum care in electronic medical records at an academic healthcare system in California (2021–2023; *N* = 5590)

Variable	Unadjusted Odds Ratio (95% CI)	*P*-Value	Adjusted Odds Ratio (95% CI)	*P*-Value
Race and Ethnicity				
Non-Hispanic Asian/Pacific Islander	1.17 (1.01–1.34)	0.034	1.18 (1.02–1.36)	0.026
Non-Hispanic Black	1.36 (1.06–1.74)	0.016	1.20 (0.93–1.54)	0.169
Non-Hispanic Other/Multiracial	1.15 (0.96–1.38)	0.129	1.13 (0.94–1.35)	0.191
Non-Hispanic White	Ref	-	Ref	-
Hispanic	1.43 (1.25–1.65)	< 0.001	1.34 (1.16–1.56)	< 0.001
Prefer English language				
Yes	Ref	-	Ref	-
No	1.01 (0.78–1.32)	0.919	0.80 (0.61–1.06)	0.125
Age at delivery				
< 25	1.25 (0.91–1.72)	0.174	1.06 (0.76–1.48)	0.727
25–34	Ref	-	Ref	-
35*+*	1.05 (0.94–1.17)	0.372	1.04 (0.93–1.17)	0.453
Parity				
Primiparous	Ref	-	Ref	-
Multiparous	0.97 (0.87–1.08)	0.588	0.95 (0.85–1.07)	0.430
Insurance at delivery				
Private	Ref	-	Ref	-
Public/None	1.38 (1.19–1.60)	< 0.001	1.28 (1.09–1.51)	0.003
Trimester prenatal care initiated				
1st	Ref	-	Ref	-
2nd	1.10 (0.93–1.30)	0.245	1.07 (0.90–1.28)	0.419
3rd	1.14 (0.93–1.40)	0.219	1.13 (0.88–1.45)	0.328
Number of prenatal care visits				
0–10	Ref	-	Ref	-
11–20	0.85 (0.67–1.07)	0.165	1.01 (0.76–1.32)	0.968
21–30	1.15 (0.90–1.48)	0.264	1.34 (1.00-1.80)	0.052
31+	1.11 (0.78–1.59)	0.565	1.17 (0.79–1.74)	0.4323
Hypertension and/or diabetes in pregnancy				
No	Ref	-	Ref	-
Yes	1.26 (1.10–1.45)	0.001	1.13 (0.98–1.31)	0.089
Depression and/or anxiety in pregnancy				
No	Ref	-	Ref	-
Yes	1.10 (0.95–1.27)	0.197	1.06 (0.91–1.23)	0.446
Delivery type				
Vaginal	Ref	-	Ref	-
Cesarean	1.32 (1.17–1.48)	< 0.001	1.24 (1.10–1.40)	< 0.001
Preterm birth				
No	Ref	-	Ref	-
Yes	1.38 (1.12–1.69)	0.002	1.26 (1.02–1.57)	0.031

## Discussion

This study leveraged EMR data from one academic healthcare system in California to identify the sociodemographic characteristics, PNC utilization, and health factors associated with completed mental health screenings in pregnancy and postpartum. Despite delivering several years after the California screening mandate, many patients did not have documented screenings during pregnancy and/or during postpartum. Race and ethnicity, preferred language, parity, insurance status, trimester of PNC initiation, number of PNC visits, delivery type, and PTB were each independently associated with perinatal mental health screenings. Findings from this study can help identify patients who are not currently benefitting from the California screening mandate and inform future efforts to fully implement the policy and achieve universal perinatal mental health screening.

In our study, 67% of patients had documented prenatal screenings in the EMR and 56% of patients who completed postpartum care had documented postpartum screenings in the EMR. A study in Minnesota similarly found that 65% of patients were screened in pregnancy and 64% were screened in postpartum, although Minnesota does not have a state law mandating screening (Sidebottom et al. 2021). In contrast, our prenatal and postpartum screening rates are lower than those found after healthcare institutions implemented universal mental health screening programs in prenatal and/or postpartum care (Avalos et al. 2016; Miller et al. 2019; Johnson et al. 2021).

We found that patients who were White (vs. API, Black, or Hispanic), multiparous, privately insured, and did not prefer English each had lower odds of completed perinatal mental health screenings than their counterparts. Our findings regarding parity and language preference align with prior literature (Sidebottom et al. 2021; Woofter et al. 2025). Although our findings regarding race and ethnicity conflict with prior studies, our patient population was more diverse than in prior literature (Sidebottom et al. 2021; Woofter et al. 2025). Also conflicting with prior findings, we found that privately insured patients had lower odds of screening than publicly insured patients (Sidebottom et al. 2021; Woofter et al. 2025). Notably, in this healthcare system, publicly insured patients generally receive prenatal and postpartum care in one clinic staffed by residents, while privately insured patients see attending physicians in their individual offices. Differences in screening practices across clinics could explain these findings.

We also found that patients who began PNC in the third trimester (vs. first trimester) or completed 10 or fewer PNC visits had lower odds of completed screenings in pregnancy than their counterparts, and patients who had vaginal deliveries and term births each had lower odds of completed screenings in postpartum than their counterparts. The findings regarding PNC initiation and total number of PNC visits align with prior literature, and may be due to these patients having more opportunities for screening (Sidebottom et al. 2021). Our findings regarding delivery type and gestational age conflict with a prior study, though that study examined screening in the hospital after delivery rather than in prenatal or postpartum care (Woofter et al. 2025).

Our findings have clinical and policy implications. Given that not all patients were screened for mental health in pregnancy and postpartum, clinicians should work to ensure universal perinatal mental health screening. Prior studies have identified barriers to perinatal mental health screening, including limited time during healthcare visits, lack of training and knowledge on perinatal mental health among OBGYNs, and concerns that patients may not be able to receive mental healthcare following screening (Palladino et al. 2011; Goldin Evans et al. 2015; Harrison 2024; Woofter et al. 2026). Since healthcare institution mandates have resulted in higher perinatal mental health screening rates (Avalos et al. 2016; Miller et al. 2019; Johnson et al. 2021; Accortt et al. 2022), institutional guidance on how to implement state screening mandates may improve screening rates even in the context of state screening mandate. Interestingly, many of the groups more frequently screened in our study (e.g., Black patients, publicly insured patients, and those with medical complications) also have higher rates of depression and anxiety (Dekel et al. 2019; Bauman et al. 2020; Yang et al. 2022; Haight et al. 2024; Bodunde et al. 2025). Still, all patients should be screened regardless of depression and anxiety risk. While evidence suggests that the California screening mandate increased screening rates (Woofter et al. 2026), our findings demonstrate that additional policy efforts are necessary to address inequities in screening. Collaboration between clinicians and policymakers may be useful to develop guidance for screening, address barriers to screening, and measure adherence to the screening mandate throughout the state.

This study has some limitations. First, the data are from one academic healthcare system in California, and findings may not be generalizable to other contexts. In particular, compared to all those giving birth in California in 2023, a smaller proportion of our patient population was Hispanic, under 35 years old, publicly insured, and multiparous, although the populations were similar in terms of trimester of prenatal care initiation, delivery type, and PTB (California Department of Public Health, Maternal, Child & Adolescent Health Division 2025). Second, it is possible that patients did complete mental health screenings on paper but that these screenings were not documented in the EMR. However, if the screening results were not properly documented in the EMR, they are not accessible for follow-up or for other clinicians to reference. Third, we are unable to determine whether patients who did not have documented screenings were offered and refused the screening or were not offered the screening at all. Fourth, we are not able to identify mental health referrals provided to patients following screening in our dataset.

Future research should build upon this study to further examine perinatal mental health screening. While most studies on screening focus on one healthcare system, studies analyzing population-based datasets would be useful. In particular, additional evidence is needed comparing screening between states with and without screening mandates or before and after state mandates were implemented. Studies using difference-in-differences methods could determine whether screening mandates had a causal effect on screening rates. Additionally, research should further investigate the patient and provider characteristics associated with perinatal mental health screening. In particular, studies should explore factors where literature is inconsistent (e.g., race and ethnicity, insurance status, maternal and infant complications). While it is important to determine screening rates and inequities in screening, it is also important to understand the quality of care provided during perinatal mental health screening. Prior work has found that patients express dissatisfaction with the perinatal mental health screening process (Hsieh et al. 2021; Webb et al. 2023; Sudhinaraset et al. 2025), suggesting an opportunity to improve screening to meet patients’ needs and preferences. Finally, given that the goal of screening is to facilitate access to mental healthcare for those with depression or anxiety symptoms, additional research is needed on referrals following screening in the context of a state screening mandate.

## Conclusion

Despite ACOG recommendations and California law, this study of electronic medical records data at one academic healthcare system in California from 2021 to 2023 found that 33% of patients were not screened for mental health in pregnancy and, of those who attended postpartum care, 44% were not screened for mental health in postpartum. In particular, patients who were White, non-English speakers, multiparous, privately insured, began prenatal healthcare in the third trimester, completed 10 or fewer prenatal healthcare visits, had vaginal deliveries, and had term births each had lower odds of completing perinatal mental health screenings than their counterparts. These findings suggest that the 2019 California perinatal mental health screening mandate has not been fully implemented. Additional efforts are necessary to achieve universal perinatal mental health screening in accordance with the California law, and these efforts should be tailored to the populations not currently screened to reduce inequities in screening.

## Electronic Supplementary Material

Below is the link to the electronic supplementary material.

Supplementary Material 1.

## Data Availability

Data are not publicly available but may be requested through the University of California Los Angeles Clinical & Translational Science Institute.
