# Supplementary material for: Who is screened for perinatal mental health? findings from an academic health system in California
Source: Arch Womens Ment Health. 2026 May 9;29(3):73. doi: 10.1007/s00737-026-01719-w (PMC13157396; doi:10.1007/s00737-026-01719-w)
Supplement: Supplementary file 1 — Supplementary Material 1. [file 737_2026_1719_MOESM1_ESM.docx]

Table 4. ICD-10 codes used to identify relevant diagnoses in electronic medical records data

| **Variable** | **ICD-10 Label** | **ICD-10 Code** |
| --- | --- | --- |
| Hypertension and/or Diabetes | Pre-existing hypertension complicating pregnancy, childbirth and the puerperium | O10.XX |
|  | Pre-existing hypertension with pre-eclampsia | O11.XX |
|  | Gestational edema and proteinuria without hypertension | O12.XX |
|  | Gestational hypertension without significant proteinuria | O13.XX |
|  | Pre-eclampsia | O14.XX |
|  | Eclampsia | O15.XX |
|  | Unspecified maternal hypertension | O16.XX |
|  | Essential (primary) hypertension | I10.XX |
|  | Diabetes mellitus due to underlying conditions | E08.XX |
|  | Type 1 diabetes mellitus | E10.XX |
|  | Type 2 diabetes mellitus | E11.XX |
|  | Other specified diabetes mellitus | E13.XX |
|  | Diabetes mellitus in pregnancy, childbirth, and the puerperium | O24.XX |
| Depression and/or Anxiety | Depressive episode | F32.XX (excluding F32.81) |
|  | Major depressive disorder, recurrent | F33.XX |
|  | Postpartum depression | F53.0 |
|  | Other anxiety disorders | F41.XX |

| **Variable** | **ICD-10 Label** | **ICD-10 Code** |
| --- | --- | --- |
| Hypertension and/or Diabetes | Pre-existing hypertension complicating pregnancy, childbirth and the puerperium | O10.XX |
|  | Pre-existing hypertension with pre-eclampsia | O11.XX |
|  | Gestational edema and proteinuria without hypertension | O12.XX |
|  | Gestational hypertension without significant proteinuria | O13.XX |
|  | Pre-eclampsia | O14.XX |
|  | Eclampsia | O15.XX |
|  | Unspecified maternal hypertension | O16.XX |
|  | Essential (primary) hypertension | I10.XX |
|  | Diabetes mellitus due to underlying conditions | E08.XX |
|  | Type 1 diabetes mellitus | E10.XX |
|  | Type 2 diabetes mellitus | E11.XX |
|  | Other specified diabetes mellitus | E13.XX |
|  | Diabetes mellitus in pregnancy, childbirth, and the puerperium | O24.XX |
| Depression and/or Anxiety | Depressive episode | F32.XX (excluding F32.81) |
|  | Major depressive disorder, recurrent | F33.XX |
|  | Postpartum depression | F53.0 |
|  | Other anxiety disorders | F41.XX |
